# 2938. Hepatitis C Virus Care Continuum in the Direct-acting Antiviral Era within the US Veterans Health Administration

**DOI:** 10.1093/ofid/ofad500.177

**Published:** 2023-11-27

**Authors:** Cara D Varley, Elliott Lowy, Emily J Cartwright, Timothy R Morgan, Karine Rozenberg-Ben-Dror, Lauren Beste, Marissa Maier

**Affiliations:** Oregon Health and Science University, Portland, Oregon; VA Puget Sound, Seattle, Washington; Emory University School of Medicine, Atlanta VA health care system, Decatur, GA; VA Long Beach Healthcare System, Long Beach, California; Veterans Administration, Woodland Hills, California; VA Puget Sound Health Care System, Seattle, Washington; VA Portland Health Care System/Oregon Health and Sciences University, Portland, OR

## Abstract

**Background:**

Estimated hepatitis C prevalence within the Veterans Health Administration is higher than the general population and is a risk factor for advanced liver disease and its associated complications. We describe the hepatitis C care continuum within the Veterans Health Administration between January 1, 2014 and December 31, 2022.

**Methods:**

Individuals in Veterans Health Administration care 2021-2022 who were eligible for direct-acting antiviral treatment between January 1, 2014 and December 31, 2022 comprised our cohort. We evaluated the proportion of Veterans who progressed through each step of the hepatitis C care continuum, and identified factors associated with initiating direct-acting antivirals, achieving sustained virologic response, and repeat hepatitis C viremia after achieving sustained virologic response.

**Results:**

We identified 133,732 Veterans with hepatitis C viremia during our study timeframe. Hepatitis C treatment was initiated in 107,134 (80.1%), with sustained virologic response achieved in 98,136 (91.6%). In those who achieved sustained virologic response, 1,097 (1.1%) had subsequent repeat viremia and 579 (52.8%) were retreated for hepatitis C. Veterans of younger ages were less likely to initiate treatment and achieve sustained virologic response, and more likely to have repeat viremia. Stimulant use and unstable housing were negatively associated with each step of the hepatitis C care continuum.

**Table 1:**
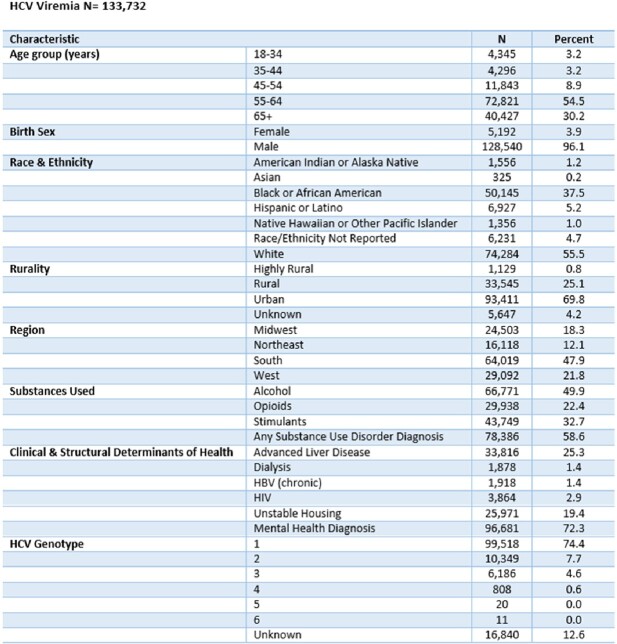
Characteristics of Veterans with HCV Viremia

**Table 2:**
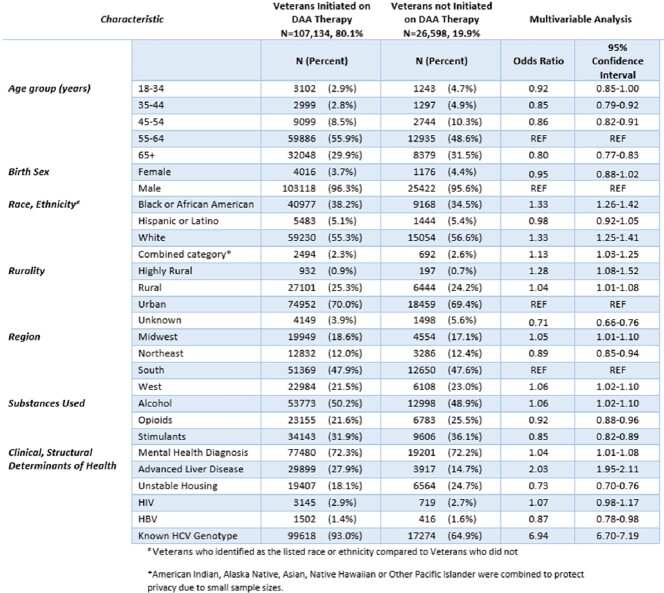
Factors Associated with DAA Treatment Initiation in those with HCV Viremia

**Figure 1:**
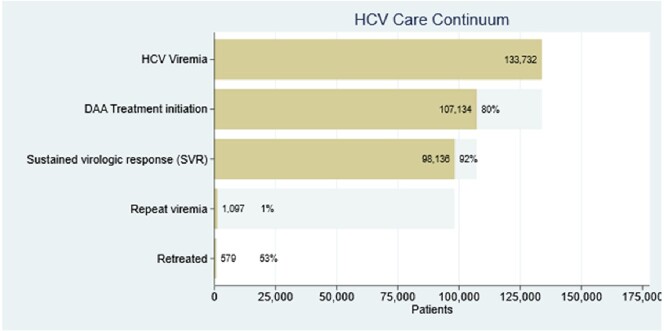
Overview of the VHA Hepatitis C Care Continuum as of December 31, 2022

**Conclusion:**

The Veterans Health Administration has treated 80% of Veterans with hepatitis C with direct-acting antiviral therapy since its approval in 2014 and achieved sustained virologic response in more than 90% of those treated. Repeat viremia is rare and is associated with younger age, unstable housing, opioid use, and stimulant use. Ongoing efforts are needed to reach younger Veterans, and Veterans with unstable housing or substance use disorders.

**Disclosures:**

**All Authors**: No reported disclosures

